# Serum Amyloid A3 Promoter-Luciferase Reporter Mice Are Useful for Early Drug-Induced Nephrotoxicity Detection

**DOI:** 10.3390/ijms25105124

**Published:** 2024-05-08

**Authors:** Ayane Kudo, Haruka Osedo, Rahmawati Aisyah, Nao Yazawa, Tolulope Peter Saliu, Kenshu Miyata, Thanutchaporn Kumrungsee, Noriyuki Yanaka

**Affiliations:** Graduate School of Integrated Sciences for Life, Hiroshima University, Hiroshima 739-8528, Japantpsa222@uky.edu (T.P.S.);

**Keywords:** nephrotoxicity, aristolochic acid, cisplatin, serum amyloid A3, *in vivo* imaging, luciferase

## Abstract

Early detection of drug-induced kidney injury is essential for drug development. In this study, multiple low-dose aristolochic acid (AA) and cisplatin (Cis) injections increased renal mRNA levels of inflammation, fibrosis, and renal tubule injury markers. We applied a serum amyloid A3 (Saa3) promoter-driven luciferase reporter (Saa3 promoter-luc mice) to these two tubulointerstitial nephritis models and performed *in vivo* bioluminescence imaging to monitor early renal pathologies. The bioluminescent signals from renal tissues with AA or CIS injections were stronger than those from normal kidney tissues obtained from normal mice. To verify whether the visualized bioluminescence signal was specifically generated by the injured kidney, we performed *in vivo* bioluminescence analysis after opening the stomachs of Saa3 promoter-luc mice, and the Saa3-mediated bioluminescent signal was specifically detected in the injured kidney. This study showed that Saa3 promoter activity is a potent non-invasive indicator for the early detection of drug-induced nephrotoxicity.

## 1. Introduction

The early detection of drug-induced nephrotoxicity and prevention of renal tubular necrosis are essential in the early phases of pharmaceutical development [1]. Animal studies have been widely conducted to examine drug-related renal toxicity because such toxicities account for approximately 30% of chemotherapeutic agent attrition during preclinical studies [2,3]. Thus, drug-induced nephrotoxicity remains an important complication that limits the efficacy of drug treatments. Various renal diseases and electrolyte disorders result from drugs used to treat malignant diseases, accounting for approximately 18–27% of all cases of acute kidney injury [1,2,3]. Cisplatin (CIS) is the most nephrotoxic chemotherapeutic drug and is typically associated with acute kidney injury in patients. Many other drugs, such as alkylating agents, antibiotics, and antimetabolites, may also exert toxic effects on renal function [1,4]. Studies on plants in the family *Aristolochiaceae* have shown potential for treating various diseases and are known to contain aristolochic acid (AA), which is associated with the development of nephropathy, i.e., AA nephropathy [5,6]. AA nephropathy is histologically characterized by a typical corticomedullary gradient of interstitial fibrosis and progressive atrophy of proximal tubules, resulting in rapid deterioration of renal function [7]. Although AA has been identified as the causative compound of these pathologies and plants known or suspected to contain AA are no longer permitted in many countries, several AA nephropathy cases are still regularly reported worldwide.

The scientific examination of animals has become a social problem. Many studies have been recently conducted with the aim of reducing the number of animal experiments to determine the pharmacological effects and toxicity tests of drugs and to establish new, non-invasive testing methods [8,9]. *In vivo* imaging is a promising technology for the non-invasive monitoring of biological processes and disease development within living animals and allows for the real-time examination of therapeutic efficacy within living animal models [10]. High-resolution bioluminescence imaging of fluorescent proteins and luciferase have recently been applied in *in vivo* research to study tumorigenesis as well as inflammatory and brain diseases [10,11]. In our previous study, we successfully established a serum amyloid A3 (Saa3) gene promoter-luciferase reporter to non-invasively monitor low-grade inflammation in white fat tissues of obese mice fed a high-fat diet and further applied it to dietary-adenine-induced renal pathologies [12,13]. Because the dietary-adenine-induced renal model shows pathologies characterized by tubulointerstitial injury, it inspired us to apply this *in vivo* Saa3-promoter bioluminescence imaging technique to monitor drug-induced renal nephropathy. Our present study suggests that this *in vivo* bioluminescence imaging could be useful for the non-invasive visualization of pathological changes in the kidney, which is characterized by drug-induced nephrotoxicity.

## 2. Results

**Figure 1:**
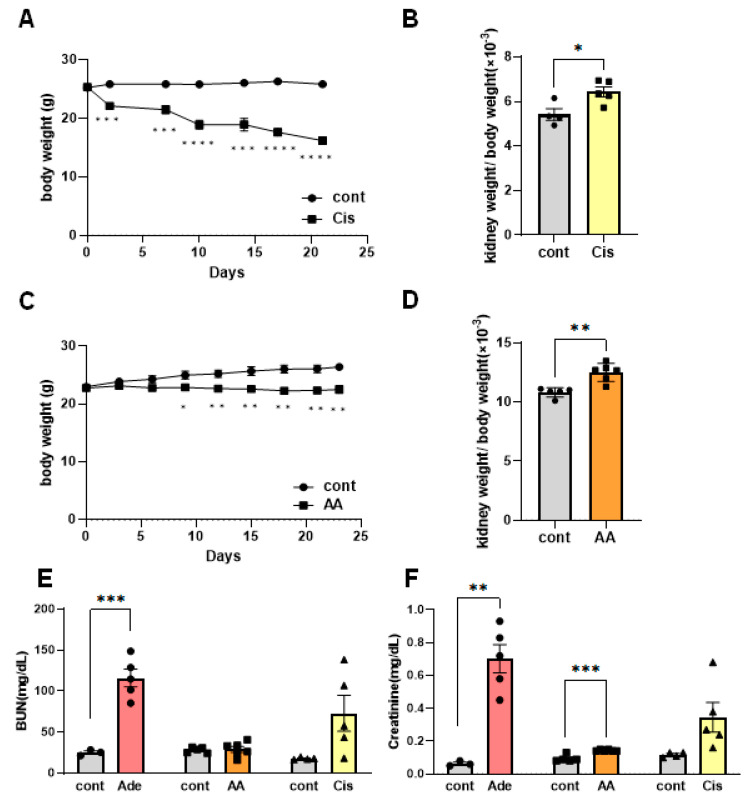
Pathological assessment of drug-induced nephrotoxicity in multiple low-dose cisplatin (CIS) and aristolochic acid (AA) models. (**A**–**D**) Body weights of CIS- and AA-injected mice decreased significantly when compared with the matched control (cont) mice. Relative kidney weights of CIS- and AA-injected mice increased significantly when compared with the matched control (cont) mice. (**E**,**F**) Plasma BUN and creatinine levels were significantly increased by an adenine diet for two weeks (Ade). AA treatment resulted in a moderate increase in blood creatinine levels. All values are expressed as means ± SEs. * *p* < 0.05, ** *p* < 0.01, *** *p* < 0.005 and **** *p* < 0.001 as determined by the Student *t* test.

CIS is a potent and widely used chemotherapeutic drug for cancer treatment with known adverse effects on organs, particularly the kidneys. Additionally, AA, a nephrotoxic agent, causes AA nephropathy, which is characterized by progressive renal fibrosis and functional decline. CIS and AA nephropathy have garnered research attention as an acute kidney injury (AKI) model for decades, and recent animal studies have focused on chronic kidney disease (CKD). Animal CKD models are widely employed, and cultured cells are expected to be used in future studies. In particular, repeated low-dose treatments with AA and CIS have been used to investigate chronic tubulointerstitial nephritis in experimental animals [14,15]. In the present study, male C57BL/6 mice were injected with 8 mg/kg CIS for three consecutive weeks or with 2.5 mg/kg AA every three days for four weeks. Repeated low-dose CIS and AA treatments led to decreased body weight and increased relative kidney weight (Figure 1A–D). To induce kidney injury, the mice were fed an adenine-containing diet for two weeks. The adenine-induced kidney disease model is well established and reliably shows pathological changes with the deposition of 2,8-dihydroxyadenine crystals in renal tubules and the accumulation of collagen, which are typical pathologies of tubulointerstitial fibrosis [13]. Blood urea nitrogen (BUN) and creatinine concentrations in mice fed an adenine diet for two weeks were significantly higher than those in the control group (Figure 1E). In contrast, repeated AA and CIS treatments did not significantly alter blood BUN concentrations (Figure 1E). Repeated low-dose CIS treatment resulted in individual differences in blood renal marker levels, whereas AA treatment resulted in moderate but significant increases in blood creatinine levels (Figure 1F). Serum creatinine level also depends on other factors such as muscle function and fasting, suggesting other side effects of AA treatment. Overall, these results suggest that CKD models treated with low-dose AA and CIS show tubulointerstitial injury during the early phase.

**Figure 2:**
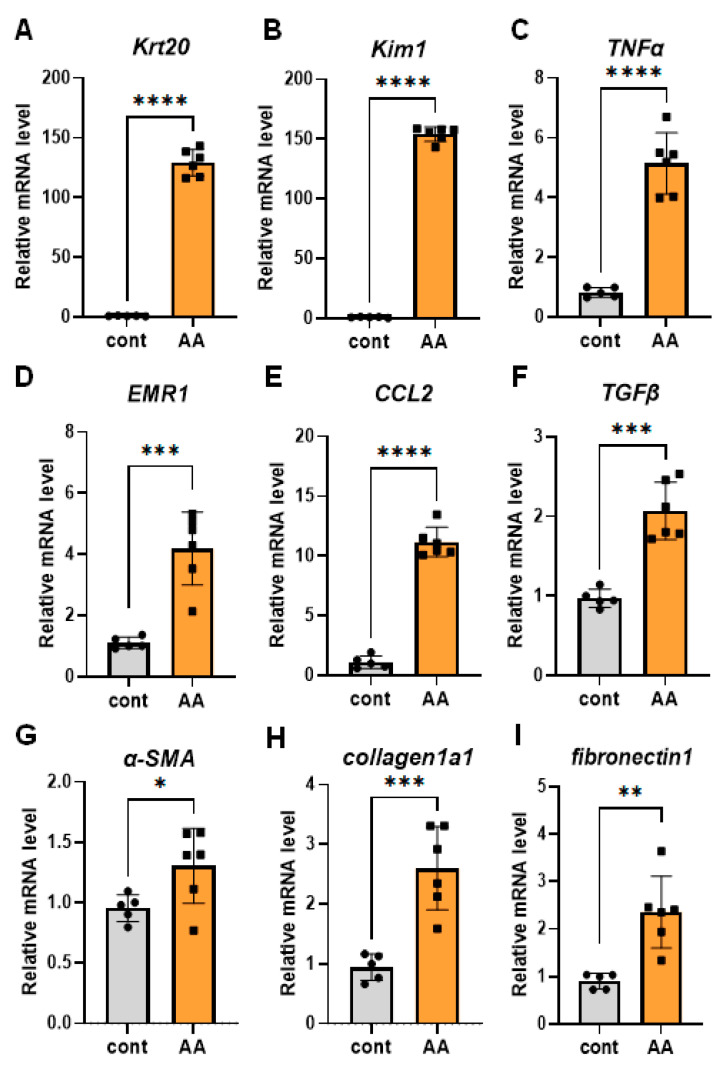
Renal fibro-inflammatory markers are upregulated in multiple low-dose aristolochic acid (AA) model. (**A**–**I**) Total RNAs in kidney tissues of multiple low-dose aristolochic acid (AA) model (*n* = 6) were isolated. The relative mRNA expression level of each gene was determined by quantitative PCR and normalized to L19 mRNA level and are presented as means ± S.E. * *p* < 0.05, ** *p* < 0.01, *** *p* < 0.001, **** *p* < 0.0001. The data are representative of two independent experiments. AA = AA nephropathy, cont = control.

**Figure 3:**
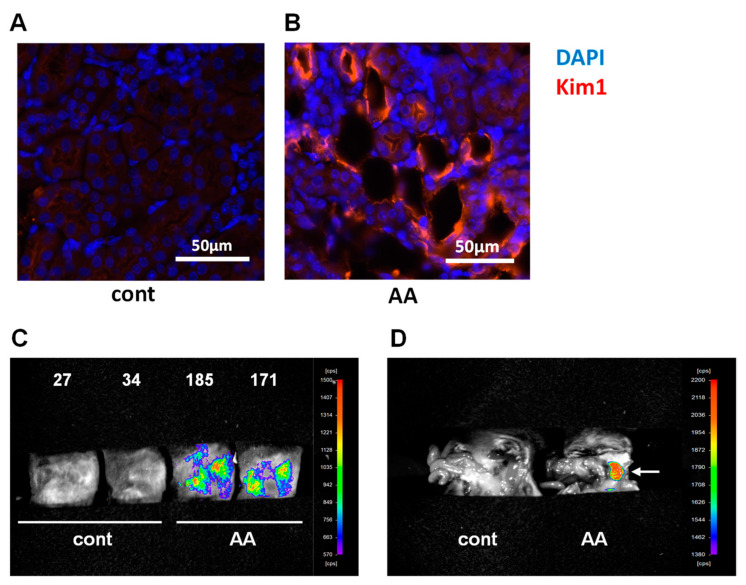
Visualization of renal pathology in multiple low-dose aristolochic acid (AA) model using Saa3 promoter-luc mice. (**A**,**B**) Representative images of injured renal tubules from kidney stained for Kim1 (red) and DAPI (blue) immunoreactivity. (**C**) *In vivo* bioluminescence imaging from the back of Saa3 promoter-luc mice shows a strong intensity of bioluminescent signal (from violet for least intense to red for most intense), reflecting kidney injury. The signal intensity was presented as counts per second (cps)/mm^2^. (**D**) Bioluminescent analysis of mouse organs exposed to bioluminescent imaging confirmed that the intense bioluminescent signal generated was specifically from the multiple low-dose aristolochic acid (AA)-induced injured kidney (the white arrow), and not from the adjacent organs of the Saa3 promoter-luc mice that were induced with HFD/multiple low-dose STZ. AA = AA nephropathy, cont = control.

Progressive proximal tubular injury (PTJ) induces the mRNA expression of kidney injury molecule-1 (Kim1) and multiple keratin (Krt) genes, including Krt20 [15]. We performed a transcriptomic analysis of PTJ and fibro-inflammatory marker genes such as tumor necrosis factor α (TNF-α), transforming growth factor β (TGF-β), collagen type I alpha1 (Col1a1), and chemokine ligand 2 (Ccl2) using qPCR on renal tissues. We found that the expression of these PTJ and fibroinflammatory markers was highly upregulated in multiple low-dose AA models (Figure 2). Kim1 is a transmembrane glycoprotein expressed by injured proximal tubular cells that has been recognized as an early, sensitive, and specific urinary biomarker of kidney injury. The immunohistological study showed that Kim1 was expressed in the proximal tubules of AA-treated mice (Figure 3A,B and Supplementary Figure S1). To monitor early renal pathology in the low-dose AA-induced CKD model, we performed *in vivo* bioluminescence imaging of Saa3 promoter-luciferase transgenic mice (Saa3 promoter-luc mice) four weeks after repeated low-dose AA treatment. As shown in Figure 3C and Supplementary Figure S2, the bioluminescence signal from the backs of AA-treated mice was stronger (from violet for the least intense to red for the most intense) than that from the kidneys of normal mice (injected with saline). To verify whether the visualized bioluminescence signal originated from the injured kidney in AA mice, we analyzed *in vivo* bioluminescence after opening the stomachs of Saa3 promoter-luc mice. These results showed that the Saa3-mediated bioluminescent signal was specifically detected in the injured kidney (white arrow, Figure 3D) but not in the adjacent organs in the AA-induced CKD model; however, the uninjured kidneys and other organs of normal mice showed no bioluminescent signal.

**Figure 4:**
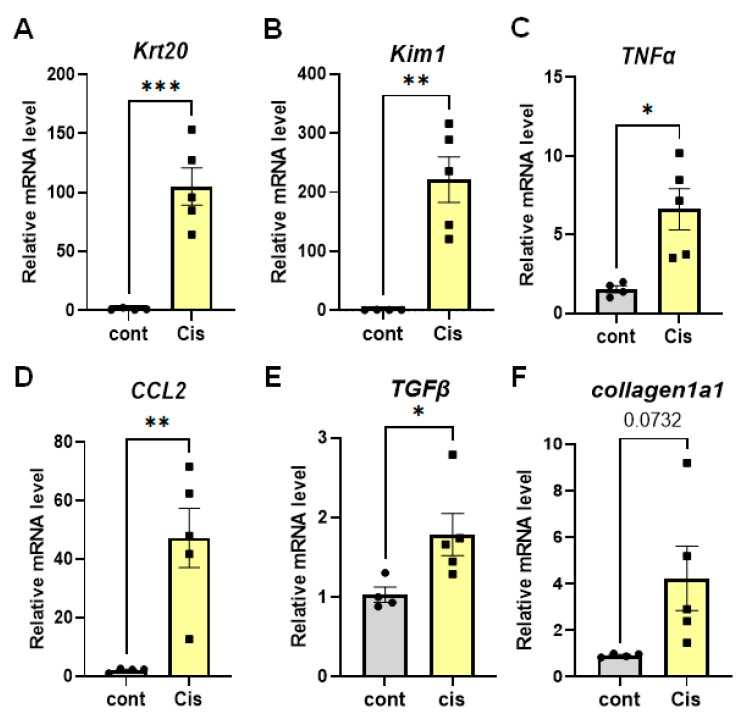
Renal fibro-inflammatory markers are upregulated in multiple low-dose cisplatin (Cis) model. (**A**–**F**) Total RNAs in kidney tissues of multiple low-dose cisplatin (CIS) model (*n* = 5) were isolated. The relative mRNA expression level of each gene was determined by quantitative PCR and normalized to L19 mRNA level and are presented as means ± S.E. * *p* < 0.05, ** *p* < 0.01, *** *p* < 0.001. The data are representative of two independent experiments. Cis = cisplatin nephropathy, cont = control.

**Figure 5:**
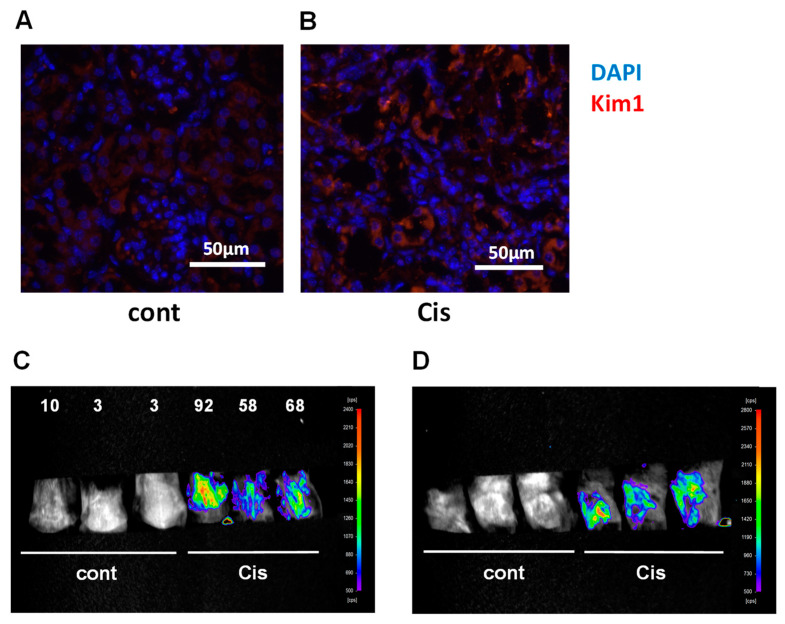
Visualization of renal pathology in multiple low-dose cisplatin (CIS) model using Saa3 promoter-luc mice. (**A**,**B**) Representative images of injured renal tubules from kidney stained for Kim1 (red) and DAPI (blue) immunoreactivity. (**C**,**D**) *In vivo* bioluminescence imaging from the back (**C**) and the side (**D**) of Saa3 promoter-luc mice shows a strong intensity of bioluminescent signal (from violet for least intense to red for most intense), reflecting kidney injury. The signal intensity was presented as counts per second (cps)/mm^2^. Cis = cisplatin nephropathy, cont = control.

Next, we analyzed the renal mRNA expression levels of PTJ, fibrosis, and inflammation markers in CIS-treated mice by qPCR. We found that the expression of PTJ and fibroinflammatory markers was high in the kidneys of mice in the multiple low-dose CIS model (Figure 4). Further immunohistological analysis showed that Kim1 protein is expressed in the proximal tubules of CIS mice (Figure 5A,B), suggesting that repeated low-dose CIS treatment induces proximal tubular injury. We further performed *in vivo* bioluminescence imaging of Saa3 promoter-luc mice three weeks after repeated low-dose CIS treatment to detect early renal pathology. As shown in Figure 5C,D and Supplementary Figure S2, the bioluminescent signal from the back and side of CIS-treated mice was stronger than that from the normal kidneys of control Saa3 promoter-luc mice injected with saline. The Saa3-promoted mediated bioluminescent signal was also detected in CIS-treated mice.

## 3. Discussion

Animal experimentation has been subject to increasing moral and social concern, and pain and distress in animals used in these experiments are recognized as important factors of animal welfare. In recent years, bioluminescence imaging with luciferase and fluorescent proteins has been developed for the non-invasive visualization of cells and biochemical events *in vivo*; thus, it is becoming an essential technique in a variety of biomedical research fields. Although many kinds of disease models, such as those for cancer metastasis and inflammatory diseases, have been developed using bioluminescence imaging and are being employed for drug screening and development *in vivo* [16,17], this technology has not yet been widely used in kidney disease models. Urinary and serum metabolites are known to be potential biomarkers for the diagnosis of kidney injury [8,9]. However, because the glomerular filtration rate is already reduced before the serum creatinine level increases, specific biomarkers are needed for the early detection of nephropathy [1,8,9]. CIS is a widely used anticancer drug for several types of carcinogenesis but is often associated with serious side effects on normal tissues, especially in kidneys. In a previous study, the acute nephrotoxicity of CIS was shown to be a typical AKI model, with a decline in renal function, tubulointerstitial fibrosis, and consequent glomerular inflammation. Repeated treatment with low-dose AA and CIS has been reported to cause long-term renal pathologies with characteristics of CKD. In this study, we performed repeated low-dose AA and CIS treatment and observed renal fibrosis and inflammatory marker expression at the RNA level, accompanied by Kim1 expression in renal epithelial cells. Moreover, we demonstrated that *in vivo* Saa3-promoter bioluminescence imaging is a sensitive and specific tool for detecting and visualizing tubulointerstitial injury in real-time in living animals with repeated low-dose AA and CIS treatment. This novel bioluminescence imaging technique has the advantage of early prediction of tubulointerstitial fibrosis, which cannot be detected or clearly distinguished by analyzing typical blood markers such as BUN and creatinine due to the low-grade toxicity to renal tubules.

In our previous study, we utilized the mouse Saa3 gene promoter to detect inflammation in the white adipose tissue of high-fat-diet-induced obese mice [12]. Saa3 is an acute-phase protein, whose expression is highly responsive to various inflammatory conditions [18,19,20,21,22]. Previous reports have demonstrated that Saa3 mRNA is highly expressed in chronic inflammatory disease models such as those for rheumatoid arthritis, atherosclerosis, and colitis [18,19,20,21,22,23]. Three CCAAT/enhancer binding protein β (C/EBPβ)-binding sites (−152, −107, and −77) are shown to be located in the mouse Saa3 gene promoter region [12,23]. C/EBPβ is reportedly involved in inflammatory processes during disease development. Inflammatory cytokines, such as IL-1 and tumor necrosis factor α (TNF-α) are shown to induce Saas mRNA expression in various diseases including kidney diseases in both patients and experimental animals [18,19,20,21,22,24]. Taken together, these observations suggest that C/EBPβ is an important target for assessing drug-induced kidney injury and that the Saa3-promoter reporter is useful for visualizing renal injury in experimental kidney disease models.

Because C/EBPβ is a transcription factor of various genes including Saa, αSMA, and Col1, recent reports have suggested an important role of C/EBPβ in tissue fibrosis [25,26,27,28]. C/EBPβ-deficient mice showed a decrease in αSMA and Col1 expression and suppression of TGF-β-induced fibroblast differentiation into myofibroblasts, which are tightly associated with extracellular matrix accumulation and tissue fibrosis [26]. Interstitial extracellular matrix accumulation is a pathologically important event in drug-induced renal injury during progressive tubulointerstitial fibrosis [29]. Interstitial myofibroblasts seen in renal fibrosis are possibly related to TGF-β1, which can activate tubular epithelial cell proliferation and induce epithelial–mesenchymal transition. In this study, we found that TGF-β1 mRNA expression level was actually increased in the renal tissues with AA or CIS treatment, suggesting that the increased bioluminescent signal from Saa3 promoter activity reflected epithelial cell proliferation and activation during tubulointerstitial fibrosis. Taken together, Saa3-promoter reporter may be used in live animals to monitor both C/EBPβ activation in kidney disease pathology and the therapeutic effects of functional foods on drug-induced kidney diseases.

## 4. Materials and Methods

### 4.1. Establishment of Saa3 Promoter-Luc Chimeric Mice

A Saa3-promoter transgenic mouse model carrying mouse Saa3 promoter region (−314/+50) upstream of the complete luciferase cDNA was generated as reported previously [12]. Heterozygous mice harboring the transgene were backcrossed at least five times with purebred C57BL/6J mice (Charles River, Kanagawa, Japan). Four-week-old male C57BL/6J mice were obtained from Charles River, Japan. Mice were housed in groups of two or three in metal cages maintained at 24 °C with a 12 h light and dark cycle (lights on, 8:00 A.M. to 8:00 P.M.). MF solid chow (Oriental Yeast, Tokyo, Japan) and deionized water were provided ad libitum. The animal study was approved by the Hiroshima University Animal Committee (Permit Number: C22-52), and the mice were maintained in accordance with the Hiroshima University Guidelines for the Care and Use of Laboratory Animals.

### 4.2. Kidney Injury Experiments

Male C57BL/6J mice (8 weeks old, Charles River), WT mice, and Saa3 promoter-luc mice (5–8 weeks old, established in this study) were housed in groups of two or three in metal cages under the conditions described above. For the adenine-induced kidney injury experiment, the adenine diet consisted of a control diet mixed with adenine (FUJIFILM Wako, Osaka, Japan) at a dose of 2 g adenine/kg diet (0.2% *w*/*w*). Male mice were intraperitoneally administered 8 mg/kg of CIS (FUJIFILM Wako, Osaka, Japan) in four consecutive weekly injections (once a week) or 2.5 mg/kg AA (MedChemExpress, Monmouth Junction, NJ, USA) every three days for four consecutive weeks.

### 4.3. In Vivo Bioluminescence Imaging Analysis

Male Saa3 promoter-luc mice were injected intraperitoneally with D-luciferin (150 mg/kg body weight, Promega) and anesthetized with isoflurane. After 5 min, Saa3-luc mice were placed in a prone position on the plate and imaged for 1 min with the camera set to the highest sensitivity level using a NightOWL II Imaging Systems LB983 (Berthold Technologies, Bad Wildbad, Germany). Photons emitted from the tissues were analyzed using Indigo *in vivo* image software (Berthold). The signal intensity was quantified as the sum of all detected photon counts per second and presented as counts per second (cps)/mm^2^. For any analysis, all images were adjusted to the same scale as the minimum and maximum luminescence intensities.

### 4.4. RT-PCR Analyses

Total RNA was isolated from the kidneys using the RNeasy Lipid Tissue Kit (Qiagen Sciences, Germantown, MD, USA). The reverse transcriptase reaction was carried out with 1 μg total RNA as a template to synthesize cDNA using ReverTra Ace qPCR RT kit (TOYOBO, Osaka, Japan) according to the manufacturer’s instructions. For quantitative PCR analysis, cDNA and primers were added to the THUNDERBIRD SYBR qPCR Mix (TOYOBO), to give a total reaction volume of 20 µL. PCR reactions were then performed using StepOnePlus (Applied Biosystems, Foster City, CA, USA). Conditions were set to the following parameters: 2 min at 95 °C, followed by 40 cycles each of 15 s at 95 °C and 1 min at 60 °C. The primers used for PCR analyses were as follows: Krt20, F, 5′-TCACCGAAGTCTGAGTTCCTC-3′, and R, 5′-CTCATTACGGCTTTGGAGACAG-3′; kim-1, F, 5′-ACATATCGTGGAATCACAACGAC-3′, and R, 5′-ACTGCTCTTCTGATAGGTGACA-3′; TNF-α, F, 5′-CCGAT GGGTTGTACCTTGTC-3′, and R, 5′-CGGACTCCGCAAAGTCTAAG-3′; EMR1, F, 5′-ATTGTGGAAGCATCCGAGAC-3′, and R, 5′-GTAGGAATCCCGCAATGATG-3′; CCL2, F, 5′-GGTCCCTGTCATGCTTCTGG-3′, and R, 5′-CCTTCTTGGGGTCAGCACAG-3′; TGFβ, F, 5′-GGCACCATCCATGACATGAA-3′, and R, 5′-TTCTCTGTGGAGCTGAAGCAAT-3′; αSMA, F, 5′-GGCTCTGGGCTCTGTA-3′, and R, 5′-CTCTTGCTCTGGGCTTCATC-3′; Col1, F, 5′-CCCAAGGAAAAGAAGC-3′, and R, 5′-ACATTAGGCGCAGGAAGGTCA-3′; fibronectin1, F, 5′-GGTGACACTTATGAGCGCCCTAAA-3′, and R, 5′-AACATGTAACCACCAGTCTCATGTG-3′; L19, F, 5′-GGCATAGGGAAGAGGAAGG-3′, and R, 5′-GGATGTGCTCCATGAGGATGC-3′. The primer sequences were designed by the Primer3 website (http://primer3.ut.ee/ accessed on 1 July 2023).

### 4.5. Immunohistochemical Analyses

Kidney tissues were fixed with 4% paraformaldehyde. Paraffin sections of 4 µm were used for immunofluorescence staining. After treatment with proteinase K solution (0.4 μg/μL) for 15 min and blocking in 5% donkey serum for 30 min at room temperature (RT), the slices were incubated with primary KIM-1 antibody (Abcam, Cambridge, UK) overnight at 4 °C. Sections were washed three times for five minutes in PBST at RT, followed by secondary antibodies incubation with anti-goat IgG Alexa Fluor 594 (1:500; Thermo Fisher Scientific, Waltham, CA, USA) at RT for 1 h. Afterward, sections were washed three times for five minutes in PBST at RT and further counterstained with 4′,6-diamidino-2-phenylindole (DAPI) for 30 min at RT. All images were captured by Olympus BX53 microscope (Olympus, Tokyo, Japan) and analyzed by Nikon Elements imaging software (Nikon, Tokyo, Japan).

### 4.6. Plasma BUN and Creatinine Analysis

Blood was collected and the plasma was immediately separated by centrifugation (10 min at 900× *g*) and stored at −30 °C. BUN and creatinine levels were determined using a Beckman Coulter AU480 analyzer (Beckman Coulter, Krefeld, Germany), which is an automated chemistry instrument used for turbidimetric, spectrophotometric, and ion-selective electrode measurements. Briefly, 200 μL plasma was used to measure these parameters according to the manufacturer’s protocol.

### 4.7. Statistical Analyses

Values are presented as means ± S.E. Statistical significance was determined by the unpaired Student’s *t*-test. For all tests, the results were considered statistically significant at *p* < 0.05.

## Data Availability

Data are contained within the article and Supplementary Materials.

## Supplementary Materials

The following supporting information can be downloaded at: https://www.mdpi.com/article/10.3390/ijms25105124/s1.

## References

[1] Van Meer L., Moerland M., Cohen A.F., Burggraaf J. (2014). Urinary kidney biomarkers for early detection of nephrotoxicity in clinical drug development. Br. J. Clin. Pharmacol..

[2] Kola I., Landis J. (2004). Can the pharmaceutical industry reduce attrition rates?. Nat. Rev. Drug Discov..

[3] Loghman-Adham M., Kiu Weber C.I., Ciorciaro C., Mann J., Meier M. (2012). Detection and management of nephrotoxicity during drug development. Expert Opin. Drug Saf..

[4] Campbell R.E., Chen C.H., Edelstein C.L. (2023). Overview of antibiotic-induced nephrotoxicity. Kidney Int. Rep..

[5] Vanherweghem J.L., Tielemans C., Abramowicz D., Depierreux M., Vanhaelen-Fastre R., Vanhaelen M., Dratwa M., Richard C., Vandervelde D., Verbeelen D. (1993). Rapidly progressive interstitial renal fibrosis in young women: As-sociation with slimming regimen including Chinese herbs. Lancet.

[6] Depierreux M., Van Damme B., Houte K.V., Vanherweghem J.L. (1994). Pathologic aspects of a newly described nephropathy related to the prolonged use of Chinese herbs. Am. J. Kidney Dis..

[7] Cosyns J.P., Jadoul M., Squifflet J.P., De Plaen J.F., Ferluga D., van Ypersele de Strihou C. (1994). Chinese herbs nephropathy: A clue to Balkan endemic nephropathy?. Kidney Int..

[8] McIlroy D.R., Wagener G., Lee H.T. (2010). Biomarkers of acute kidney injury: An evolving domain. Anesthesiology.

[9] Sieber M., Hoffmann D., Adler M., Vaidya V.S., Clement M., Bonventre J.V., Zidek N., Rached E., Amberg A., Callanan J.J. (2009). Comparative analysis of novel noninvasive renal biomarkers and metabonomic changes in a rat model of gentamicin nephrotoxicity. Toxicol. Sci..

[10] Gross S., Gammon S.T., Moss B.L., Rauch D., Harding J., Heinecke J.W., Ratner L., Piwnica-Worms D. (2009). Bioluminescence imaging of myeloperoxidase activity *in vivo*. Nat. Med..

[11] Wessels J.T., Busse A.C., Mahrt J., Dullin C., Grabbe E., Mueller G.A. (2007). *In vivo* imaging in experimental preclinical tumor research–A review. Cytom. Part A.

[12] Sanada Y., Yamamoto T., Satake R., Yamashita A., Kanai S., Kato N., van de Loo F.A., Nishimura F., Scherer P.E., Yanaka N. (2016). Serum amyloid A3 gene expression in adipocytes is an indicator of the interaction with macrophages. Sci. Rep..

[13] Kumrungsee T., Kariya T., Hashimoto K., Koyano T., Yazawa N., Hashimoto T., Sanada Y., Matsuyama M., Sotomaru Y., Sakurai H. (2019). The serum amyloid A3 promoter-driven luciferase reporter mice is a valuable tool to image early renal fibrosis development and shows the therapeutic effect of glucosyl-hesperidin treatment. Sci. Rep..

[14] Huang L., Scarpellini A., Funck M., Verderio E.A., Johnson T.S. (2013). Development of a chronic kidney disease model in C57BL/6 mice with relevance to human pathology. Nephron Extra.

[15] Ma Z., Hu X., Ding H.-F., Zhang M., Huo Y., Dong Z. (2022). Single-nucleus transcriptional profiling of chronic kidney disease after cisplatin nephrotoxicity. Am. J. Pathol..

[16] Hochgräfe K., Mandelkow E.-M. (2012). Making the brain glow: *In vivo* bioluminescence imaging to study neurodegeneration. Mol. Neurobiol..

[17] Luker K.E., Luker G.D. (2010). Bioluminescence imaging of reporter mice for studies of infection and inflammation. Antivir. Res..

[18] Ye R.D., Sun L. (2015). Emerging functions of serum amyloid A in inflammation. J. Leukoc. Biol..

[19] Eklund K.K., Niemi K., Kovanen P.T. (2012). Immune functions of serum amyloid A. Crit. Rev. Immunol..

[20] Sorić Hosman I., Kos I., Lamot L. (2021). Serum amyloid A in inflammatory rheumatic diseases: A compendious review of a re-nowned biomarker. Front. Immunol..

[21] Chami B., Hossain F., Hambly T.W., Cai X., Aran R., Fong G., Vellajo A., Martin N.J., Wang X., Dennis J.M. (2019). Serum amyloid A stimulates vascular and renal dysfunction in apolipoprotein E-deficient mice fed a normal chow diet. Front. Immunol..

[22] Hartigh L.J.D., Wang S., Goodspeed L., Ding Y., Averill M., Subramanian S., Wietecha T., O’Brien K.D., Chait A. (2014). Deletion of serum amyloid A3 improves high fat high sucrose diet-induced adipose tissue inflammation and hyperlipidemia in female mice. PLoS ONE.

[23] Geurts J., Vermeij E.A., Pohlers D., Arntz O.J., Kinne R.W., Berg W.B.v.D., van de Loo F.A. (2011). A novel Saa3-promoter reporter distinguishes inflammatory subtypes in experimental arthritis and human synovial fibroblasts. Ann. Rheum. Dis..

[24] Djurec M., Graña O., Lee A., Troulé K., Espinet E., Cabras L., Navas C., Blasco M.T., Martín-Díaz L., Burdiel M. (2018). Saa3 is a key mediator of the protumorigenic properties of cancer-associated fibroblasts in pancreatic tumors. Proc. Natl. Acad. Sci. USA.

[25] Hu B., Wu Z., Jin H., Hashimoto N., Liu T., Sem H., Phan S.H. (2004). CCAAT/enhancer-binding protein isoforms and the regu-lation of alpha-smooth muscle actin gene expression by IL-1 beta. J. Immunol..

[26] Hu B., Wu Z., Nakashima T., Phan S.H. (2012). Mesenchymal-specific deletion of C/EBPβ suppresses pulmonary fibrosis. Am. J. Pathol..

[27] Houglum K., Buck M., Adir V., Chojkier M. (1994). LAP (NF-IL6) transactivates the collagen alpha 1(I) gene from a 5’ regulatory region. J. Clin. Investig..

[28] Hu B., Ullenbruch M.R., Jin H., Gharaee-Kermani M., Phan S. (2006). An essential role for CCAAT/enhancer binding protein β in bleomycin-induced pulmonary fibrosis. J. Pathol..

[29] Herrera-Pérez Z., Gretz N., Dweep H. (2016). A comprehensive review on the genetic regulation of cisplatin-induced nephrotoxi-city. Curr. Genom..

