# Supplementary material for: Serum Amyloid A3 Promoter-Luciferase Reporter Mice Are Useful for Early Drug-Induced Nephrotoxicity Detection
Source: Int J Mol Sci. 2024 May 8;25(10):5124. doi: 10.3390/ijms25105124 (PMC11120996; doi:10.3390/ijms25105124)
Supplement: Supplementary file 1 [file ijms-25-05124-s001.zip › ijms-2934254-supplementary.pdf]

Serum Amyloid A3 Promoter-Luciferase Reporter Mice Are Useful for Early Drug-Induced Nephrotoxicity Detection

Ayane Kudo, Haruka Osedo, Rahmawati Aisyah, Nao Yazawa, Tolulope Peter Saliu, Kenshu Miyata, Thanutchaporn Kumrungsee, and Noriyuki Yanaka

Supplementary figures:

Supplementary figure S1

Supplementary figure S2

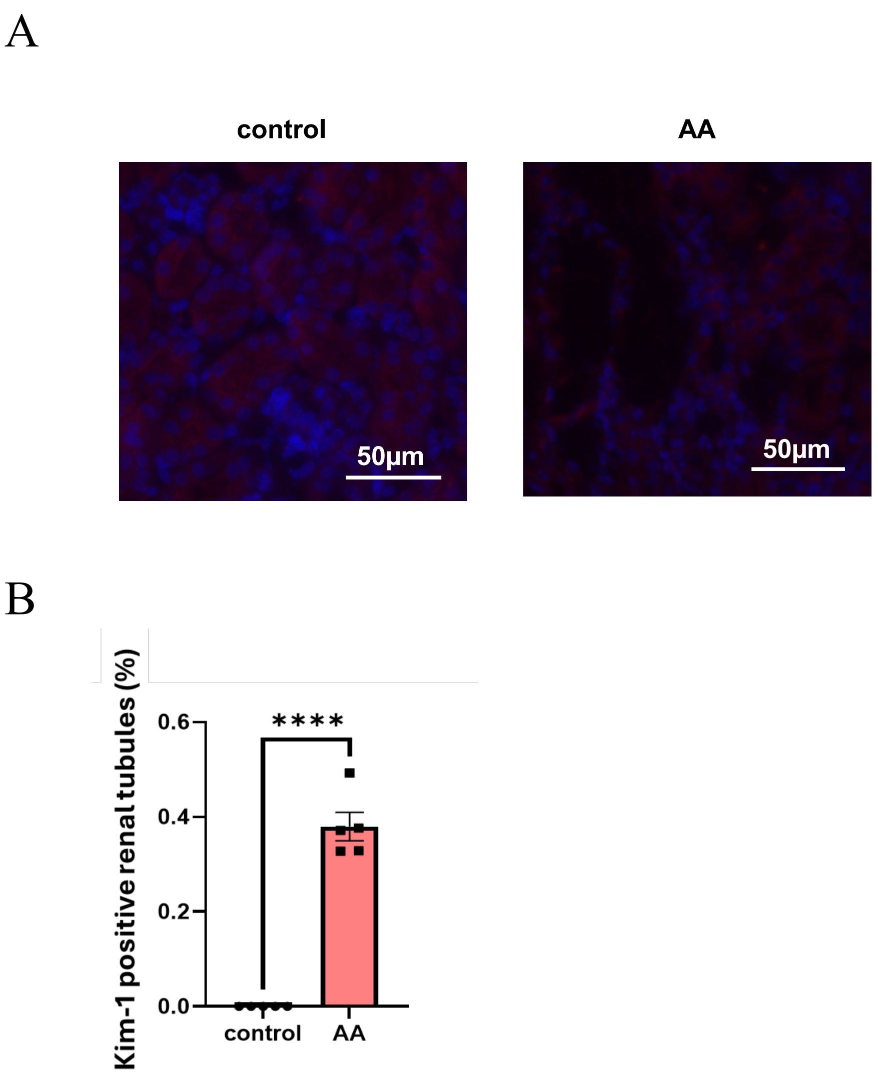

Figure S1. (**A**), Representative images of injured renal tubules from kidney stained without primary Kim1 antibody. Kidney tissues were fixed with 4% paraformaldehyde. Paraffin sections were incubated without primary KIM-1 antibody overnight at 4 °C, followed by antibodies incubation with anti-goat IgG Alexa Fluor 594 (1:500; Invitrogen) at RT for 1 h and further counterstained with 4',6-diamidino-2-phenylindole (DAPI) for 30 min at RT (blue). All images were captured by Olympus BX53 microscope (Olympus, Tokyo, Japan) and analyzed by Nikon Elements imaging software (Nikon, Tokyo, Japan). (**B**), The quantification of Kim-1 positive renal tubule numbers. Kidney tissues were fixed with 4% paraformaldehyde. Paraffin sections were incubated with KIM-1 antibody overnight at 4 °C, followed by antibodies incubation with anti-goat IgG Alexa Fluor 594 (1:500; Invitrogen) at RT for 1 h and further counterstained with DAPI for 30 min at RT. All images were captured by Olympus BX53 microscope (Olympus) and analyzed by Nikon Elements imaging software (Nikon). The number of Kim-1 positive renal tubule was determined manually by blind counting under the microscope. All values are expressed as mean ± S.E. Student's *t*-test, ****$p < 0.0001$. AA = AA nephropathy.

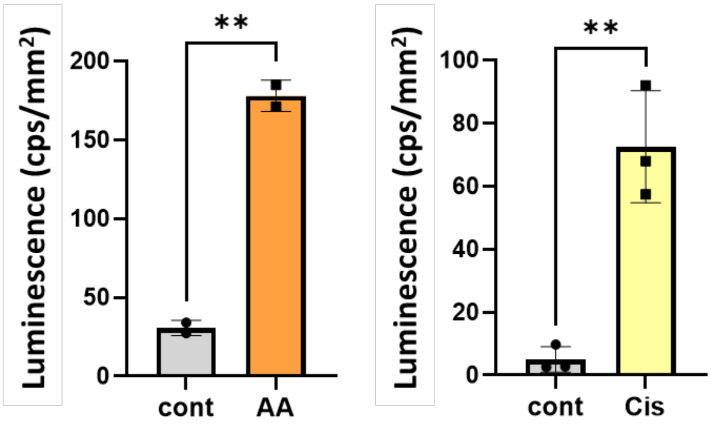

Figure S2. Male Saa3 promoter-luc mice were injected intraperitoneally with D-luciferin (150 mg/kg body weight, Promega) and anesthetized with isoflurane. Saa3 promoter-luc were imaged by NightOWL II Imaging Systems LB983 (Berthold Technologies, Bad Wildbad, Germany). Photons emitted from tissues were analyzed using Indigo in vivo image software (Berthold). Signal intensity was quantified as the sum of all detected photon counts per second and presented as count/sec (cps)/mm^2. All values are expressed as mean ± S.E. Student's *t*-test, $**p < 0.01$. cont = control, AA = AA nephropathy, Cis = cisplatin nephropathy.
